# Partial Purine Nucleoside Phosphorylase Deficiency Helps Determine Minimal Activity Required for Immune and Neurological Development

**DOI:** 10.3389/fimmu.2020.01257

**Published:** 2020-06-30

**Authors:** Eyal Grunebaum, Nicholas Campbell, Matilde Leon-Ponte, Xiaobai Xu, Hugo Chapdelaine

**Affiliations:** ^1^Division of Immunology and Allergy, Hospital for Sick Children, Toronto, ON, Canada; ^2^Developmental and Stem Cell Biology Program, Hospital for Sick Children, Toronto, ON, Canada; ^3^Department of Medicine, Centre Hospitalier de I'Universite de Montreal, and Montreal Clinical Research Institute, Montreal, QC, Canada

**Keywords:** deficiency, gene therapy, mutations, partial, purine nucleoside phosphorylase

## Abstract

**Introduction:** Complete or near complete absence of the purine nucleoside phosphorylase (PNP) enzyme causes a profound T cell immunodeficiency and neurological abnormalities that are often lethal in infancy and early childhood. We hypothesized that patients with partial PNP deficiency, characterized by a late and mild phenotype due to residual PNP enzyme, would provide important information about the minimal PNP activity needed for normal development.

**Methods:** Three siblings with a homozygous *PNP* gene mutation (c.769C>G, p.His257Asp) resulting in partial PNP deficiency were investigated. PNP activity was semi-quantitively assayed by the conversion of [14C]inosine in hemolysates, mononuclear cells, and lymphoblastoid B cells. PNP protein expression was determined by Western Blotting in lymphoblastoid B cells. DNA repair was quantified by measuring viability of lymphoblastoid B cells following ionizing irradiation.

**Results:** A 21-year-old female was referred for recurrent sino-pulmonary infections while her older male siblings, aged 25- and 28- years, did not suffer from significant infections. Two of the siblings had moderately reduced numbers of T, B, and NK cells, while the other had near normal lymphocyte subset numbers. T cell proliferations were normal in the two siblings tested. Hypogammaglobulinemia was noted in two siblings, including one that required immunoglobulin replacement. All siblings had typical (normal) neurological development. PNP activity in various cells from two patients were 8–11% of the normal level. All siblings had normal blood uric acid and increased PNP substrates in the urine. PNP protein expression in cells from the two patients examined was similar to that observed in cells from healthy controls. The survival of lymphoblastoid B cells from 2 partial PNP-deficient patients after irradiation was similar to that of PNP-proficient cells and markedly higher than the survival of cells from a patient with absent PNP activity or a patient with ataxia telangiectasia.

**Conclusions:** Patients with partial PNP deficiency can present in the third decade of life with mild-moderate immune abnormalities and typical development. Near-normal immunity might be achieved with relatively low PNP activity.

## Introduction

**Figure 1:**
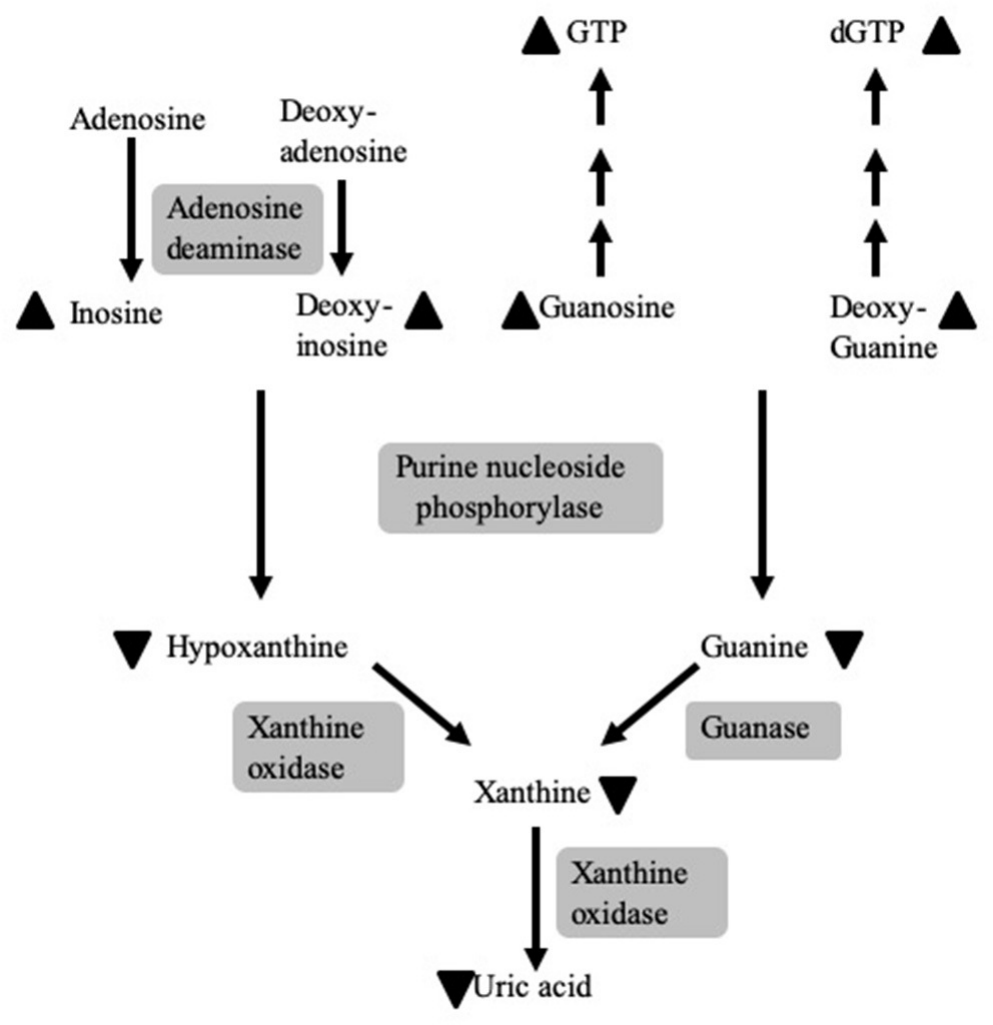
The role of PNP in purine metabolism and the consequences of PNP deficiency. PNP reversibly catalyzes the phosphorolysis of inosine, deoxy-inosine, guanosine, and deoxy-guanosine. PNP deficiency causes the accumulation (depicted by upward pointing arrowheads) of the enzymes' substrates and their phosphorylated derivatives, GTP and dGTP, while preventing the generation (depicted by downward pointing arrowheads) of hypoxanthine, guanine, xanthine, and, subsequently, uric acid. Enzymes are noted by a gray background.

Purine nucleoside phosphorylase (PNP) is a 32 kiloDalton (kDa) enzyme that reversibly catalyzes the phosphorolysis of inosine, deoxy-inosine, guanosine, and deoxy-guanosine (Figure 1) (1). Complete or near complete absence of PNP causes the accumulation of the enzyme's substrates in the blood and urine, while preventing the generation of the enzyme's products, hypoxanthine, xanthine, and, eventually, uric acid (2, 3). Additionally, there is an increase in the concentrations of the phosphorylated derivatives of guanosine and deoxy-guanosine, GTP and dGTP, respectively. The accumulation of dGTP in the mitochondria of cells interferes with DNA maintenance and repair (2). The latter leads to enhanced apoptosis of cells undergoing rapid replication or cells exposed to oxidative and irradiation stress (2, 4), similar to the decreased survival of cells with a mutated *ataxia telangiectasia* gene (5).

Autosomal recessive defects in PNP enzymes were recognized in the 1970's among patients with a profound T cell immunodeficiency (6). Similar to another inherited purine defect, adenosine deaminase (ADA) deficiency, progressive abnormalities in B and Natural Killer (NK) lymphocyte subsets, as well as in the myeloid lineage, are also identified in PNP-deficient patients (4, 7). PNP-deficient patients often suffer from bacterial, viral, fungal, or opportunistic infections in the first year of life (8, 9). The immune dysregulation associated with PNP deficiency also leads to autoimmunity, resulting in hematological cytopenia and frequent malignant transformation (10, 11). Non-infectious dysfunction, particularly neurological abnormalities such as ataxia and spasticity, are frequently reported in PNP-deficient patients (12) and in mice lacking the PNP enzyme (13). These anomalies could be due to the ubiquitous nature of the PNP enzyme and the diverse biological roles of PNP and purine metabolites.

Antimicrobial treatment and prophylaxis can prevent some of the infections caused by PNP deficiency, yet most patients die from infections, autoimmunity, or malignancy in infancy and early childhood (14–16). Allogeneic hematopoietic stem cell (HSC) transplantations can correct the immune deficiency and possibly halt or even improve the neurological deficits. However, transplant-related complications are still frequent and typical (normal) development is not always achieved, possibly because of incomplete donor chimerism that limits the ability of PNP-proficient hematopoietic cells to restore purine metabolism (8, 12, 17–21). Moreover, even when donor chimerism is achieved, neurological deficits might persist. These anomalies could possibly be due to irreversible pre-transplantation damage or the need for PNP activity within neuronal cells, as also seen in some patients with ADA deficiency after HSC transplantations (22, 23).

Alternative treatments that are being explored for PNP deficiency include injections of a PNP enzyme replacement with human PNP fused to a protein transduction domain (TATPNP) (24) or gene therapy with the PNP gene delivered *ex-vivo* into autologous PNP-deficient HSC (25). Although these treatments are currently not available for clinical use, determining the minimal PNP activity required for correcting the immune and neurological abnormalities associated with PNP deficiency would be an important step for the design and assessment of potential future clinical treatments.

We hypothesized that patients with partial PNP deficiency, characterized by a late and mild phenotype due to residual PNP enzymes, would provide valuable information regarding the PNP levels that would be sufficient for normal or near normal immune and neurological development.

Here we describe three siblings with PNP deficiency who were identified in adulthood with mild-moderate immune abnormalities and typical neurological function.

## Materials and Methods

### Patients

Three siblings with a homozygous c.769C>G mutation in the *PNP* gene were studied. All studies were in accordance with the Research Ethics Boards of the Center Hospitalier de I'Universite de Montreal, Montreal, Quebec and the Hospital for Sick Children, Toronto, Ontario.

### Immune Evaluations

Flow cytometry was used to enumerate lymphocyte subsets and the percentages of 24 V-beta families among CD4^+^ and CD8^+^ T cells with the IOTest® Beta Mark T-cell repertoire assay (Beckman Coulter, IND, US), as previously described (26). Proliferations of peripheral blood mononuclear cells (PBMC) were determined following 4 days' stimulation with phytohemagglutinin (PHA) by 3[H]thymidine incorporation. Stimulation Index (SI) was calculated as the ratio of 3[H]thymidine incorporation into cells with or without stimulation. The upper limit of SI for patients with combined immune deficiency is 241.3. Proliferations of CD4^+^ T singlet viable cells following five days' stimulation with antiCD3 (5 μg/ml) and soluble antiCD28 (2 μg/ml) were also measured by flow cytometry using the intracellular violet dye dilution (CellTrace™ Violet by Thermofisher).

### PNP Enzyme Activity

PNP activity, expressed as a percentage of a same day healthy control, was measured semi-quantitively as previously described by conversion of [8-14C]inosine to hypoxanthine, separated by thin-layer chromatography (27) with slight modifications, as results were not normalized to protein or hemoglobin content of the samples. PNP activity was measured in hemolysates containing predominantly erythrocytes, in PBMC and in EBV transformed lymphoblastoid B cells, established as previously described (28). PNP activity *in vivo* was assessed by measuring uric acid in the blood, guanosine, and deoxyguanosine in dried blood spots, as recently described (29), and PNP substrates in the urine using liquid chromatography–tandem mass spectrometry.

### PNP Protein Expression

PNP expression was determined by Western blotting, as previously described (30), in lysates of 5 × 10^6^ lymphoblastoid cells from patient II.1, a patient with absent PNP activity (c.172C>T, pArg57Ter and 285+1 G>A), previously reported (31), an ADA-deficient patient (c. 646 G>A, p. Gly216Arg and c.955delGAGAA), and a healthy control. Additionally, 100 ng of TATPNP with a molecular weight of ~34 kDa (30) and molecular weight markers were also blotted. Following the separation of the proteins and transfer to a nitrocellulose membrane (Biorad), 1:1,000 diluted monoclonal mouse-anti human PNP antibody (LS-C137543 from Life-Span Bio Inc, Seattle CA) was added. Proteins were detected with the appropriate secondary antibody and enhanced Amersham ECL Western blotting detection reagents (GE Healthcare, Chicago, IL). Membranes were reblotted with beta-actin. The intensity of the PNP and beta-actin bands were determined by the IMAGEJ open platform software and the ratio between the bands was calculated.

### DNA Repair

DNA repair was determined by measuring the survival of lymphoblastoid cells (0.5 × 10^6^/*ml*) 3 days after 10 Gy ionizing irradiation, as previously described (32). The survival of cells established from patients II.1 and II.2, the patient with absent PNP activity described above, two unrelated PNP-proficient controls, and a patient with a mutated *ataxia telangiectasia* gene (c. 4642delGATA and c. 5932G>T, p Glu1978Ter) were compared. Survival fraction (SF) was calculated by dividing the number of viable cells following irradiation by the initial number of viable cells in non-irradiated cultures. Statistical differences in SF were determined by 2-way Anova and considered significant if *p* < 0.05.

## Results

### Patients

**Figure 2:**
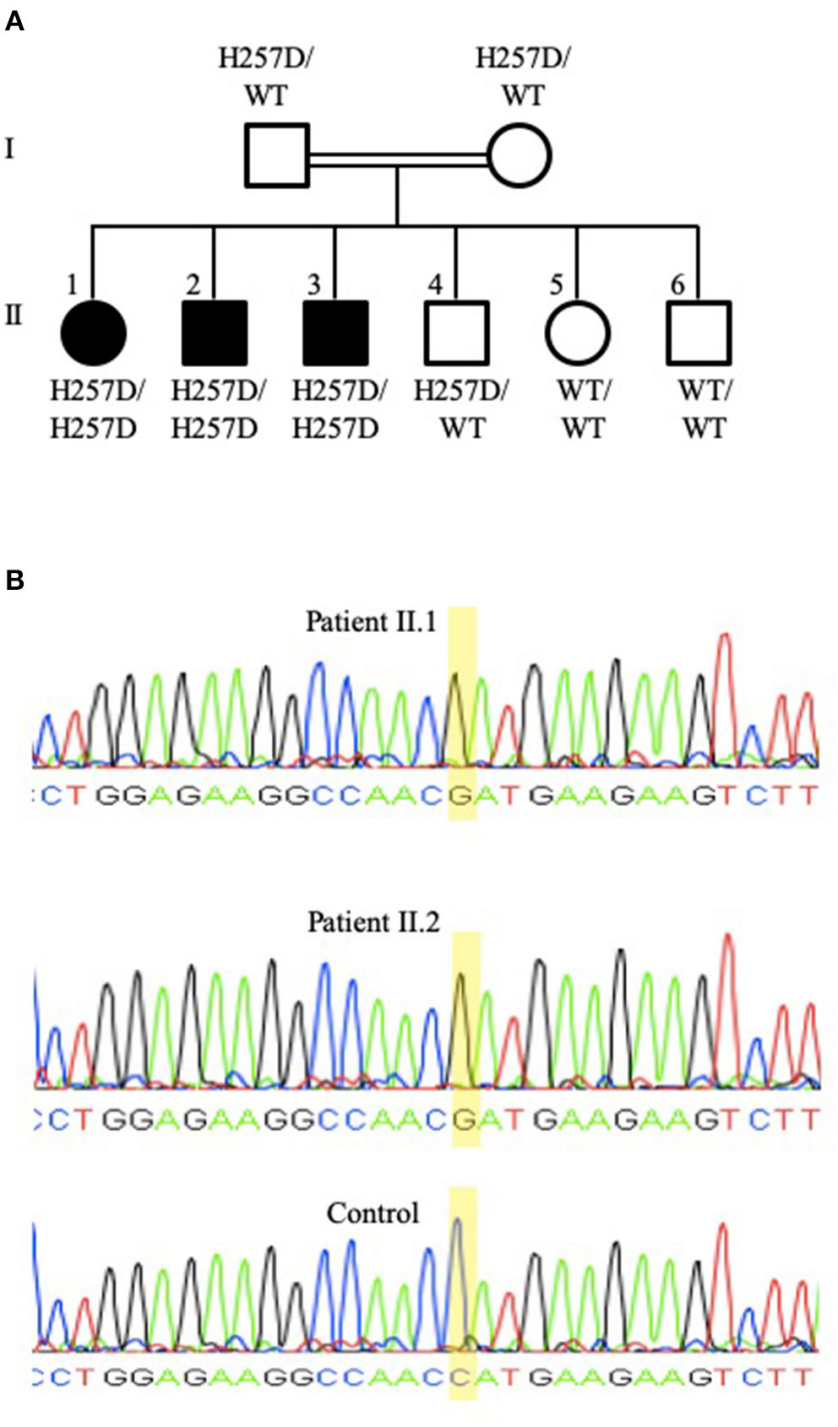
Family pedigree and *PNP* gene sequencing of patients II.1 and II.2. **(A)** Family pedigree demonstrating the three siblings (II.1, II.2, and II.3) that are homozygous for the *PNP* gene mutation putatively causing p.His257Asp (depicted as H257D), while the parents (I.1 and I.2) and an additional sibling (II.4) were heterozygous for the mutated and for the wild-type (depicted as WT) alleles. **(B)** Sequencing of the *PNP* gene Sanger sequencing of the *PNP* gene from patients II.1 (upper panel) and II.2 (middle panel) demonstrating a G nucleotide at position 769, while a healthy control (bottom panel) has a C nucleotide at this position. Sequencing was performed with the forward primer TTACAGGTGTGAACCACTGC and the reverse primer GAAGAAAGTGGGAAAGGTGA.

Patient II.1 (family pedigree depicted in Figure 2A), a 21-year-old female university student, was referred for an evaluation of recurrent sino-pulmonary infections and persistent lymphopenia. The patient had pneumonia at 8 and 20 years of age, chickenpox at 4 years of age (she did not receive the varicella vaccine), and shingles at 19 years of age. She also suffered from pan-sinusitis and underwent nasal polypectomy at 20 years of age. The patient had an episode of transient pneumonia-associated neutropenia that resolved after a short course of granulocyte colony-stimulating factor. She had received the measles, mumps, and rubella vaccine with no complications and had not experienced oral thrush or other fungal infections. She was the youngest of six children of consanguineous parents. The patient's physical examination, including a detailed neurological assessment, was normal. The patient developed asthma with no indication for bronchiectasis in radiological imaging of her chest. Following the diagnosis of the immunodeficiency and subsequently of hypogammaglobulinemia, the patient was treated with prophylactic trimethoprim-sulfamethoxazole and immunoglobulin infusions with a temporary improvement of her sinus disease. DNA isolated from the patient's PBMCs and tested with a commercial panel (Blueprint Genetics®) for 232 genes associated with primary immunodeficiency (PID) revealed a homozygous variant c.769C>G in exon 6 of the *PNP* gene. The presence of the mutation was subsequently confirmed by Sanger Sequencing (Figure 2B, upper panel). The variant was previously reported in a patient with PNP deficiency with compound heterozygous mutations (33). The variant putatively replaced a moderately conserved histidine at position 257 with aspartic acid (p.His257Asp). *In silico* analysis with PolyPhen, SIFT, and MutationTaster predicted the variant to be deleterious and disease-causing. Disease-causing mutations in other potential PID genes were not identified. The homozygous mutation was also identified in siblings II.2 (Figure 2B middle panel) and confirmed by Sanger sequencing. The patients' parents and sibling II.4 were heterozygous for the variant.

Patient II.2, a 25-year-old male, had uncomplicated chickenpox at 2 years of age (he had not received the varicella vaccine) and did not report any significant infections apart from a knee wound infection following a scooter accident at 16 years of age and warts at 25 years of age. He was working as an operator of heavy machinery. Patient II.3, a 28-year-old male, had few upper respiratory tract infections in early childhood, severe chickenpox at 5 years of age, shingles at 6.5 years of age, and pneumonia at 10 years of age. He was reported to have difficulties with fine motor skills and co-ordination as a teenager. Both patients II.2 and II.3 had not suffered from autoimmunity or malignancy and their physical examination, including a detailed neurological assessment, was normal. The patients' parents and the three remaining siblings had not suffered from recurrent infections, autoimmunity, or malignancies, although the father (I.1) was investigated for an inflammatory lung nodule.

### Immune Evaluations

**Figure 3:**
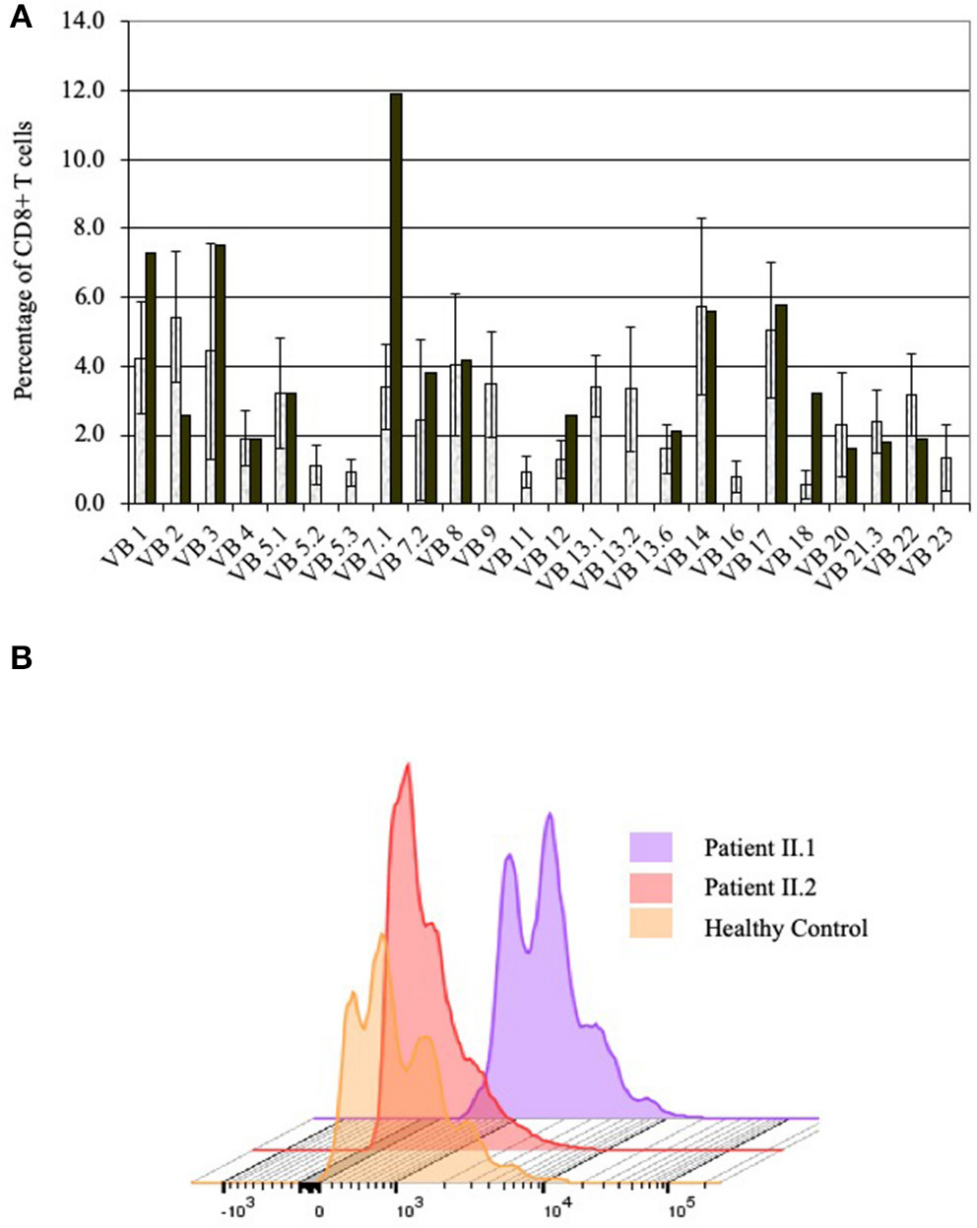
Immune evaluations in patients with partial PNP deficiency. **(A)** CD8^+^ T cells diversity in patient II.1. T cell diversity was determined by flow cytometry as the percentage of CD3^+^CD8^+^ T cells expressing one of 24 TCR V-beta chains. Diversity of patient II.1 is depicted by black bars, while the mean and 1 standard deviation of healthy donors are represented in the open bars. **(B)** Proliferation of CD4^+^ T cells of patients with partial PNP deficiency. The response of CD4^+^ T cells from patient II.1 (light violet) and II.2 (light pink) after stimulation with antiCD3 and soluble antiCD28 for 5 days was measured by flow cytometry dilution of intracellular Violet dye and compared with same day healthy control (yellow).

The numbers of lymphocytes were near normal in patient II.2, and moderately reduced in patients II.1 and II.3, with normal white blood cells and neutrophil numbers in the three siblings (Table 1). Patients II.1 and II.3 also had moderately reduced numbers of CD4^+^ and CD8^+^ T cells, CD19^+^ B cells, and CD3^−^CD16^+^CD56^+^ NK cells in comparison to age-matched healthy controls (34), while the number of lymphocyte subpopulations of patient II.2 were normal or near normal. Both patients II.1 and II.2 had reduced numbers of naïve T cells, while T cell receptor excision circles were not detected. The percentages of the CD3^+^CD4^+^CD25^+^CD127^Low^ regulatory T cells were normal. The diversity of patient II.1 T cells was restricted, particularly for CD8^+^ T cells, where 8 of 24 V-beta families were absent (Figure 3A). Patients II.1 and II.2 PBMC demonstrated normal SI to PHA. Similarly, patients II.1 and II.2 CD4^+^ T cells demonstrated normal proliferation, as determined by flow cytometry analysis of violet dye dilution (Figure 3B). Patient II.1 initially had elevated IgG at 28.5 g/l, which decreased to 4.2 g/l following effective treatment of her sinusitis. Patient II.3 also had hypogammaglobulinemia, while patient II.2 had normal IgG. Patient II.1 did not have antibodies to tetanus or diphtheria, despite previous vaccinations, although patients II.1 and II.2 had antibodies to EBV. Patient II.3 had antibodies to hepatitis B following vaccination. Patient II.1 also had elevated anti-nuclear antibodies at 1:640, while in patients II.1 and II.3 antibodies to double-stranded DNA and extractable nuclear antigens were not detected and C3 and C4 complement levels were normal.

**Table 1:** Laboratory evaluations of the patients with partial PNP deficiency.

	**II.1**	**II.2**	**III.3**	**Healthy controls**
Age (years) tested	21	25	28	
White blood cells (10^6^/*liter*)	4,930	6,310	7,800	4,500–11,000
Neutrophils (10^6^/liter)	3,900	5,600	7,030	1,300–7,500
Lymphocytes (10^6^/liter)	**300**	**920**	**400**	1,000–4,100
CD3^+^CD4^+^ (10^6^/liter)	**213**	538	**230**	450–1,990^*^
CD3^+^CD8^+^ (10^6^/liter)	**5**	184	**50**	190–940^*^
CD19^+^ (10^6^/liter)	**36**	**96**	**20**	140–600^*^
CD3^−^CD16^+^CD56^+^ (10^6^/liter)	**21**	80	**50**	70–680^*^
CD4^+^CD45RA^+^ (%)	**5**	**19.4**	ND	34.6
CD4^+^CD45RO^+^ (%)	**76.5**	**42.6**	ND	10.8
CD8^+^CD45RA^+^ (%)	**1.5**	**3.2**	ND	28.0
CD8^+^CD45RO^+^ (%)	**5.7**	**7.7**	ND	8.5
T cell receptor excision circles (copy number/3 microliter)	**Not detected**	**Not detected**	ND	ND
CD3^+^CD4^+^CD25^+^CD127^Low^ Regulatory T cells (%)	8.7%	10.6%	ND	4.0–17.0
Stimulation index	297.4	455.0	ND	>241.3
Albumin g/l	41	45	**52**	36–45
IgG (g/l)	15.4 → **4.2**	11.2	**5.4**	7.67–15.9
IgM (g/l)	0.92	2.62	**0.24**	0.37–2.86
IgA (g/l)	1.86	0.47	1.00	0.6–3.56
IgE kU/l	**145**	96	<18	<100
Anti-tetanus antibodies	**Not detected**	1.34	ND	>0.1
Anti-diphtheria antibodies	**Not detected**	0.14	ND	>0.1
Antibodies to varicella zoster	ND	Present	ND	Present
Antibodies to EBV	Present	Present	ND	Variable
Isohemagglutinins	**Anti-A 1:16** Anti-B 1:64	ND	ND	Anti-A 1:128 Anti-B 1:64
C3 g/l	1.22	ND	1.21	0.85–2.00
C4 g/l	0.16	ND	0.3	0.13–0.67

**Bold**, abnormal results; ND, Not done.

* *2.5–97.5 percentile for age matched healthy control*.

### PNP Evaluations

PNP deficiency typically causes blood and urine accumulation of the enzyme's substrates inosine, guanosine, deoxy-inosine, and deoxy-guanosine as well as reduced or absent generation of uric acid. Patients II.1 and II.2 had normal blood concentration of guanosine and deoxy-guanosine. Similarly, uric acid in the blood of II.1, II.2, and II.3 were in normal range. In contrast, PNP substrates, undetected in the urine of healthy PNP-proficient individuals, were elevated in the urine of the patients II.1, II.2, and II.3 (Table 2).

**Table 2:** PNP activity in the patients with partial PNP deficiency.

	**II.1**	**II.2**	**II.3**	**Healthy control**
Blood guanosine (μmol/l)	1.1	2.8	ND	0.5–7.4
Blood deoxy-guanosine (μmol/l)	0.8	1.5	ND	0.2–3.5
Blood uric acid (mmol)	274	215	373	167–409
Urine inosine (μmol/l)	**31**	**9**	**35**	0
Urine guanosine (μmol/l)	**4**	**1**	**8**	0
Urine deoxy-inosine (μmol/l)	**20**	**11**	**24**	0
Urine deoxy-guanosine (μmol/l)	**5**	**3**	**7**	0
PNP activity^*^ in hemolysates %	**10.7**	**8.9**	ND	50–100
PNP activity^*^ in lymphocytes %	**8.8**	**9.1**	ND	50–100
PNP activity^*^ in lymphoblastoid cells %^**^	**8.4** **±** **2.8**	**10.5** **±** **1.3**	ND	50–100

**Bold**, abnormal results; ND, Not done.

* PNP activity was measured semi-quantitively.

** *Results are the mean ± standard deviation of 3 independent experiments*.

PNP activity in hemolysates is typically absent or markedly reduced in PNP-deficient patients. In contrast, it was 182 and 166 nmol/min/ml in patients II.1 and II.2, respectively (10.7 and 8.9%, respectively, of a same day healthy control) (Table 2). ADA activity in the hemolysates of patients II.1 and II.2, measured to ensure integrity of the blood sample, was normal at 104 and 75 nmol/min/ml, respectively. Similar to the results of the hemolysates, PNP activity in PBMC from patients II.1 and II.2 was reduced at 0.13 and 014 nmol/min/10^6^ cells, respectively (8.8 and 9.1% of a same day healthy control, respectively). Because of the potential variability in measurements of single patient's samples, we also measured PNP activity in the patients' cell lines, which allowed repeated assessments and comparison to multiple controls simultaneously (Table 2). PNP activity in the patients II.1 and II.2 was 8.4 ± 2.8% and 10.5 ± 1.3% of healthy controls (3 independent experiments).

**Figure 4:**
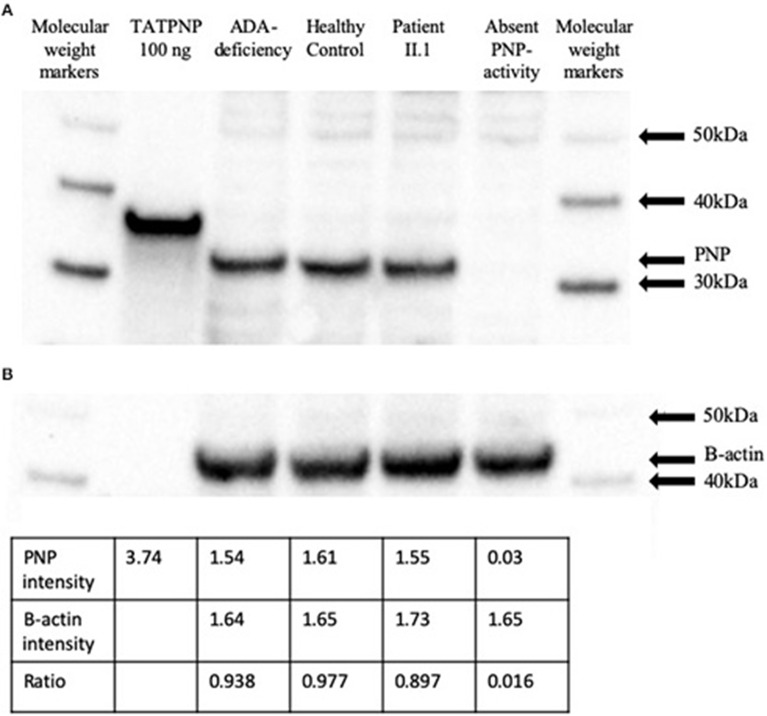
PNP protein expression in cells from a patient with partial PNP deficiency. **(A)** Western blotting of EBV transformed lymphoblastoid cells from patient II.1, a patient with complete PNP deficiency, a PNP-proficient healthy control, and an ADA-deficient patient. TATPNP and PNP that have molecular weights of 34 and 32 KDa, respectively, and molecular weight markers were also blotted. **(B)** Semi-quantitative assessment of PNP protein in EBV transformed lymphoblastoid cells from the patients and controls described above were calculated by comparison to beta-actin band intensity and TATPNP using the IMAGEJ open platform software.

Western blotting was performed on EBV transformed lymphoblastoid cell lysates to evaluate the effects of the *PNP* gene mutation on its protein expression (Figure 4A). While PNP protein was not detected in lymphoblastoid cells established from a patient with absent PNP, an expected 32 kDa band was visible in lanes correspondent to lysates obtained from a PNP-proficient healthy control and an ADA-deficient patient. Similarly, a 32 kDa band was detected in lymphoblastoid cells obtained from patient II.1. Also, an expected 34 kDa band was observed for the lane loaded with TATPNP. Semi-quantitative assessment, by comparison of PNP to beta-actin ratio and band intensity for TATPNP, suggested that the amount of PNP in the cells of patient II.1 was ~50 ng and similar to that in PNP-proficient cells (Figure 4B).

### Sensitivity of Lymphoblastoid Cells to Irradiation

**Figure 5:**
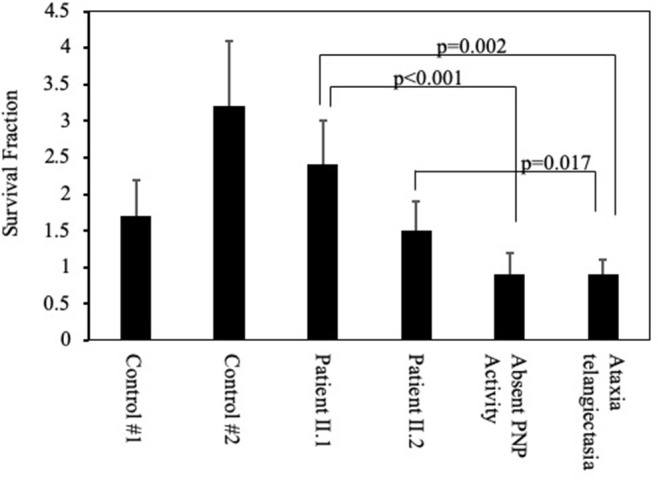
Sensitivity of cells from patients with partial PNP deficiency to irradiation. Viability of lymphoblastoid cells from the patients II.1 and II.2, a patient with complete absence of PNP activity, a patient with ataxia telangiectasia, and two unrelated PNP-proficient controls was determined 3 days after irradiation with 10 Gy. The Survival Fraction (SF) was calculated by dividing the number of viable cells following irradiation by the number of viable cells in non-irradiated cultures. Statistical differences in SF were determined by 2-way Anova.

Purine metabolites are important for DNA replication and repair, and abnormal purine homeostasis can increase sensitivity of cells to ionizing irradiation (4). Accordingly, the viability of lymphoblastoid cells was determined 3 days after irradiation with 10 Gy in comparison with the initial number of cells before irradiation (Figure 5). As expected, lymphoblastoid cells obtained from a patient with a mutated *ataxia telangiectasia* gene and a patient with a complete absence of PNP activity were particularly sensitive to irradiation, resulting in a significantly reduced (p < 0.001) SF compared to controls. In contrast, cells established from patients II.1 and II.2 had SF that were not statistically different from PNP-proficient controls but significantly increased compared to cells from patients with ataxia telangiectasia and absent PNP activity.

## Discussion

Here we report the identification and characterization of three siblings in their third decade of life with a homozygous His257Asp *PNP* gene mutation resulting in partial PNP deficiency. PNP deficiency has been previously reported in fewer than 80 patients, with many presenting with severe immunodeficiency in infancy and only a few surviving into adulthood (35). Among the longer surviving PNP patients is a 21-year-old pregnant female with absent PNP who remained largely infection-free since her presentation at 3 years of age, yet was treated with prophylactic antibiotics and immunoglobulins (9). She also suffered from ataxia, spasticity, and significant intellectual delay. Another PNP-deficient patient was healthy until 6 years of age when she developed a severe autoimmune cytopenia followed by sinusitis and a fatal chickenpox episode at 11 years of age (35). A third patient, with “late-onset” PNP deficiency with very low PNP activity, suffered from recurrent respiratory infections resulting in bronchiectasis and was treated with intravenous immunoglobulins until HSC transplantation at 13 years of age (36). In contrast, among the three siblings described here, one patient had no infections and a near normal immune evaluations at 25 years of age, while the other two patients had only moderate infections and laboratory abnormalities that did not require significant medical interventions in the initial two decades of life. Also, none of the siblings suffered from the autoimmunity or malignancies that are common among PNP-deficient patients (35). Thus, to the best of our knowledge, the siblings reported here with partial PNP deficiencies are the oldest individuals to be identified with abnormal PNP activity and with the mildest phenotype, further expanding the age of presentation and clinical manifestations associated with this rare defect.

The patients described here illustrate an additional aspect in the diagnosis of PNP deficiency. Hypouricemia has been considered a possible clue to the diagnosis of PNP deficiency among patients with PID (37). Yet similar to a recent report that found normal uric acid in five of seven PNP-deficient individuals (33), the patients described here had normal blood uric acid, and two of them also had normal PNP substrates in their blood, possibly because of the residual PNP activity. Hence, rather than blood uric acid and purine metabolites, the measurement of PNP substrates in the urine might be a more sensitive assay for abnormal PNP function, although such a test is not readily available in many centers.

Among the siblings carrying the same PNP gene mutation, there were some discrepancies in the severity of infections and immune abnormalities. One sibling had recurrent sino-pulmonary infections and required immunoglobulin replacement, while the other two siblings were asymptomatic for many years. Although PNP activity was measured only in the cells of patients II.1 and II.2, the similar normal blood uric acid and increased urine PNP substrates observed in the three siblings suggest that all have similar PNP enzyme activity. Hence, it is likely that the phenotypic differences between the siblings are not related to the measured level of enzyme activity, but rather to yet unrecognized extrinsic or intrinsic factors, as demonstrated already for other monogenic primary immune deficiencies (38).

To further investigate the biological significance of the residual PNP activity found in the patients, we tested *ex vivo* functions that are dependent on PNP, including cells' proliferation and sensitivity to irradiation (2, 4, 39). Remarkably, the proliferation of patients' II.1 and II.2 T cells to stimulation was normal, which might also explain their ability to survive varicella, which has commonly been reported as a devastating infection among PNP-deficient patients (8, 17, 35, 36, 40). Similarly, the survival of lymphoblastoid B cells from the patients II.1 and II.2 to ionizing irradiation was normal and significantly increased in comparison to a patient with absent PNP (4). Importantly, the ability to maintain protective immunity with only 8–11% PNP activity suggests this might be a sufficient threshold activity required for treating PNP deficiency with current allogenic HSC or future autologous HSC transplants and enzyme replacement therapies.

Neurological deficits, particularly tonus and motor abnormalities, occur in many PNP deficient patients (1). In contrast, the three siblings reported here had typical (normal) neurological development, possibly because of the residual PNP activity in their neuronal cells. Accelerated apoptosis of PNP-deficient neuronal cells has been suggested as the cause of these abnormalities (13). Further evidence for this hypothesis is the correlation found here between the neurological status and the resistance of cells to irradiation. Cells from a patient with a mutated *ataxia telangiectasia* gene and from a patient with absent PNP activity, who had suffered from ataxia and delayed motor skills, demonstrated markedly decreased survival following irradiation. In contrast, cells from the neurologically-intact patients described here had normal survival following irradiation. This finding further supports our previous observation that achieving 10–20% of normal PNP activity in brain cells could prevent the neurological abnormalities affecting PNP-deficient mice (13).

Information obtained from PNP-deficient patients with unique mutations has been instrumental for designing PNP transition-state analog inhibitors (41). Such PNP inhibitors have been investigated as potential treatments for the uncontrolled T and B cell proliferation seen in leukemia, autoimmune diseases, transplant rejection, and graft vs. host disease (41, 42). The histidine at position 257 of the PNP molecule directly interacts with the enzyme's substrates, yet there have been conflicting data regarding its role in PNP catalytic mechanism (43, 44), hence the interest in the biochemical effects of mutated His257 *in vivo*. A His257Asp mutation has been reported previously in a 19-year-old PNP-deficient patient together with a 172C>T variant that putatively caused a premature stop (33). The compound PNP mutations led to normal blood uric acid despite absent enzyme activity, however additional clinical and immunological data were not provided, thereby limiting the ability to appreciate the impact of the His257 mutation. As the patients described here were homozygous for the mutation, we were able to better elucidate the biochemical and biological impact of His257. The normal uric acid and PNP substrates found in the blood of the patients suggests that PNP maintained a significant catalytic activity *in vivo*. Similarly, the 8–11% PNP enzyme activity in the cells of patients II.1 and II.2 are concordant with a previous report of 5% catalytic activity following site directed mutagenesis of the His257, albeit to alanine rather than to the aspartic acid reported here (43). Notably, the results of the semi-quantitative PNP assay performed here were not normalized to protein content, limiting the comparison of our findings to those of other labs. Nevertheless, the similar values obtained using different cell types and healthy controls, as well as the practically identical PNP protein content in the Western blotting, support the accuracy of the enzyme activity assay used here. Taken together, these results indicate that the His257 is important, yet dispensable, for PNP activity.

In conclusion, here we expand the phenotype of inherited defects associated with PNP deficiency and demonstrate that patients with partial PNP deficiency can present in adulthood with only mild-moderate immune abnormalities and a normal neurological development. We also show that near-normal immunity and typical development can be achieved with 8–11% PNP activity, which suggests that such activity might be the minimum required for future therapeutic interventions.

## Data Availability Statement

All datasets generated for this study are included in the article/supplementary material.

## Ethics Statement

The studies involving human participants were reviewed and approved by Research Ethics Boards of the Centre Hospitalier de I'Universite de Montreal, Montreal, Quebec and the Hospital for Sick Children, Toronto, Ontario. The patients/participants provided their written informed consent to participate in this study.

## Author Contributions

EG and HC conceptualized this study and designed the experiments. HC, NC, and EG managed the patients. All authors contributed to writing, reviewing the manuscript, and data visualization. XX and ML-P conducted the experiments.

## Conflict of Interest

The authors declare that the research was conducted in the absence of any commercial or financial relationships that could be construed as a potential conflict of interest. The reviewer MH declared a past co-authorship with one of the authors EG to the handling editor.

## Abbreviations

ADA: Adenosine deaminase
AT: Ataxia telangiectasia
HSC: Hematopoietic stem cells
KDa: kiloDaton
NK: Natural killer
PBMC: Peripheral blood mononuclear cells
PNP: Purine nucleoside phosphorylase
SI: Stimulation index
SF: survival fraction
PID: Primary immunodeficiency
TATPNP: TAT fused to PNP.
